# Dietary Factors and Interventions for Cognitive Impairment and Dementia

**DOI:** 10.3390/nu17020210

**Published:** 2025-01-08

**Authors:** Alice L. Dawson, Auriel A. Willette

**Affiliations:** 1 Lighthouse Institute, Chestnut Health Systems, Chicago, IL 60610, USA; a.l.dawson.sciences@gmail.com; 2 Department of Neurology, Rutgers University, New Brunswick, NJ 07101, USA

Nutrition is an important lifestyle factor that can reduce the risk of future cognitive impairment and various neurodegenerative disorders, such as Alzheimer’s disease (AD) and AD-related dementias (ADRDs). Dietary interventions may be a useful approach to slow down cognitive decline or cognitive impairment across the spectrum for AD and other dementias. To date, in older adults, certain nutrients like folate, vitamin E, and Ω-3 fatty acids and food groups like seafood, vegetables, and fruits have shown promising associations with cognitive outcomes or results in randomized clinical trials. This Special Issue, “Dietary Factors and Interventions for Neurodegenerative Diseases”, focuses on how diet or nutritional components are related to cognitive decline, cognitive impairment, and the risk or age of onset of dementia. This Special Issue features a total of five papers, all of which were original research articles. One article is currently under review.

For the Li et al. article on B vitamins and brain aging, the goal was to regress B6, B12, and folate on 21 spatially orthogonal resting state neural networks using UK Biobank data [1]. A higher intake of vitamins B6 and B12 was related to less connectivity in several neural networks, including the posterior portion of the Default Mode Network (B6, beta = −0.011, *p* < 0.001; B12, beta = −0.002, *p* < 0.001). Lower connectivity in the Default Mode Network is a reliable early risk factor for Alzheimer’s disease [2]. By contrast, greater folate consumption was related to more connectivity to some of these same networks, including the posterior Default Mode Network (beta value = 0.0002, *p* < 0.001). Importantly, for participants with a mother or father who had Alzheimer’s disease (i.e., family history positive, or FH+), there was a 9.09-fold increase in posterior Default Mode Network connectivity versus FH− participants (beta estimate 0.000291 vs. 0.000032).

For the more recent Li et al. paper [3] regarding coffee and tea consumption, regressions were carried out between standard coffee, black tea, or green tea and seven resting state neural networks. Briefly, greater coffee or green tea intake was related to more functional connectivity in visual networks, and coffee specifically for motor, cingulate, and prefrontal networks. Higher black tea consumption, by contrast, was related to less connectivity in networks, including a memory consolidation network. Apolipoprotein ε4 (*APOE*4) carriers, the strongest genetic risk factor for Alzheimer’s disease [4], showed less functional connectivity in the memory consolidation network versus non-*APOE*4 carriers (*APOE*4: β = −0.0313, *p* < 0.001; non-*APOE*4: β = 0.0302, *p* < 0.001). A similar pattern was noted for people with versus without a family history of Alzheimer’s disease.

In their current study, Pocevičiūté, Wennström, and Ohlsson [5] examined the effects of an Okinawa-based Nordic (O-BN) diet among 30 adults with type 2 diabetes (T2D) on plasma glial fibrillary acidic protein (GFAP) levels, which researchers used as a proxy measure of neuroinflammation. After 3 months of a regimented O-BN diet, older (58 ± 8 years) adults (*n* = 30) with T2D had significantly reduced plasma GFAP levels compared to baseline levels (*p* = 0.048). However, after distinguishing between those who had T2D complications (+compl) and those that did not (−compl), *only* the −compl T2D participants had significant reductions in plasma GFAP after 3 months of the O-BN diet. After the 3-month period, participants were told that they could return to unrestricted food consumption. At a 4-month follow-up, plasma GFAP levels did not significantly differ from baseline plasma GFAP levels (*p* = 0.370), including when examined among those with or without T2D complications (*p* = 0.293 vs. p = 0.776). Interestingly, plasma GFAP levels were directly correlated with plasma levels of the neurodegeneration biomarker NfL, as well as with C-peptide and triglycerides at baseline. Finally, the researchers found no association between psychological well-being and plasma GFAP levels at any timepoint in the study.

In their recent AD mouse model study, Martens and their colleagues [6] found significant effects on mouse cognitive symptoms and biomarkers after supplementation of their diet with seaweed extracts, namely a lipid extract of seaweed *Himanthalia elongata* (*H. elongata*) and a supercritical fluid (SCF) extract of *S. fusiforme*, which lacks the inorganic arsenic usually found in *S. fusiforme* and its extracts. In this APPswePS1∆E9 (AD) mouse model, both extracts showed promise in preventing or postponing cognitive decline. In particular, the transgenic mice that had a diet supplemented with *H. elongata* showed prevention of cognitive decline in a 12-week treatment pre/post using established animal model tests of object memory, spatial memory, and spatial working memory (*p* = 0.0260, *p* = 0.0147 and *p* = 0.0212, respectively) compared to wild-type (WT) mice. Although the mice supplemented with *S. fusiforme* trended towards reduced cognitive decline, pre/post memory tests did not reach significance. Immunohistochemistry (ICH) analysis showed reduced glial markers with either form of supplementation. The researchers also performed RNA sequencing on the hippocampus of 1-week treated AD transgenic mice. This sequencing indicated that *H. elongata* extracts affected hippocampal calcium, acetylcholine, and synaptogenesis signaling pathways, among others. Interestingly, *H. elongata*’s effects were differentiated between transgenic mice and WT mice, the latter of which showed effects on pathways related to endoplasmic reticulum stress and Huntington’s disease, which suggests a disease-specific mechanism of *H. elongata* in these mouse models. These researchers noted that desmosterol, an endogenous LXR agonist with anti-inflammatory properties, increased in AD transgenic mice receiving either form of seaweed supplementation in their diet, which the researchers theorized may be a mechanism through which increased LXR production indirectly (1) leads to the prevention of the cytotoxic accumulation of intracellular lipids and (2) leads to the transrepression of NF-_K_B pathways through the suppression of inflammation. Although the amyloid plaque burden remained unaffected in both treatment groups, these findings suggest a high potential for *H. elongata* in particular as a mechanism to prevent or prolong the decline associated with AD, through reductions in tauopathy in disease-associated regions.

Finally, Umeda et al. [7] examined the potential medicinal properties of the leaves of *Acorus tatarinowii* Schott and *Acorus gramineus* Solander, which are used in traditional Chinese medicine for amnestic symptoms, on three mouse strains, Tau784, APP23, A53T, which are models of frontotemporal dementia (*FTD*), AD, and dementia with Lewy bodies (*DLB*), respectively. The researchers tested three different preparation methods, including hot water extract, extraction residue, and non-extracted simple crush powder, and focused specifically on the *Acorus tatarinowii* rhizome (ATR), *Acorus gramineus* rhizome (AGR), *Acorus tatarinowii* leaf (ATL), and *Acorus gramineus* leaf (AGL). Among the preparation methods, the simple crush powder was overall the most effective across the models. Immunohistochemistry revealed significantly improved pathologies in the hippocampi of APP23 AD-model mice receiving crush powder compared to their Tg littermates and significantly rescued cognition to levels similar to that of non-Tg mice. In a particularly striking result, the researchers found that crush powder was significantly neuroprotective against Aβ and α-synuclein pathologies and rescued cognition in both FTD and AD mouse models to levels similar to those of their non-Tg littermates. In a mouse model of DLB (Huα-Syn (A53T mice)), A53T mice had significantly lower levels of BDNF, which were restored by crush powder supplementation over 1 month, specifically in pathology-associated regions. Although α-synuclein levels were not completely rescued in A53T mice, this BDNF restoration indicated reductions in specific toxic oligomers to levels similar to those in non-Tg mice.

These results from Umeda et al. [7] strongly support the hypothesis that crush powder forms of these leaves are a potential dietary source for dementia prevention and treatment across multiple dementia pathologies, with a strong effect on tauopathy in mouse models of FTD, DLB, and AD. These leaves show promise as cost-effective and environmentally friendly preventative medicine and treatment option for various forms of dementia. However, given that these leaves contain over 150 compounds, some of which become toxic at high concentrations, these leaves, particularly in their crush powder form, require further examination in non-human primate animal models or others to determine their safety for long-term consumption.

Collectively, these articles evidence the complex interplay between diet and ADRD risk. Several naturally occurring compounds are highlighted which show great promise as potential therapeutic agents in the prevention and/or treatment of diverse dementia pathologies, signaling that these occurring compounds deserve much more attention and examination in future research.

## References

[1] Li T., Steibel J.P., Willette A.A. (2024). Vitamin B6, B12, and Folate’s Influence on Neural Networks in the UK Biobank Cohort. Nutrients.

[2] Mevel K., Chételat G., Eustache F., Desgranges B. (2011). The default mode network in healthy aging and Alzheimer’s disease. Int. J. Alzheimer’s Dis..

[3] Li T., Fili M., Mohammadiarvejeh P., Dawson A., Hu G., Willette A.A. (2024). Associations of Coffee and Tea Consumption on Neural Network Connectivity: Unveiling the Role of Genetic Factors in Alzheimer’s Disease Risk. Nutrients.

[4] Michaelson D.M. (2014). APOE ε4: The most prevalent yet understudied risk factor for Alzheimer’s disease. Alzheimer’s Dement..

[5] Poceviciute D., Wennstrom M., Ohlsson B. (2024). Okinawa-Based Nordic Diet Decreases Plasma Glial Fibrillary Acidic Protein Levels in Type 2 Diabetes Patients. Nutrients.

[6] Martens N., Zhan N., Yam S.C., Leijten F.P.J., Palumbo M., Caspers M., Tiane A., Friedrichs S., Li Y., van Vark-van der Zee L. (2024). Supplementation of Seaweed Extracts to the Diet Reduces Symptoms of Alzheimer’s Disease in the APPswePS1ΔE9 Mouse Model. Nutrients.

[7] Umeda T., Sakai A., Shigemori K., Nakata K., Nakajima R., Yamana K., Tomiyama T. (2024). New Value of Acorus tatarinowii/gramineus Leaves as a Dietary Source for Dementia Prevention. Nutrients.

